# Five-Year Incidence of Urinary Incontinence in 68,066 Breast Cancer Patients Followed in Gynecology Practices in Germany

**DOI:** 10.3390/diseases14010003

**Published:** 2025-12-24

**Authors:** Lara Ilona Becker, Karel Kostev, Matthias Kalder

**Affiliations:** 1 Marburg University, Department of Gynecology and Obstetrics, University Hospital, Baldingerstraße, 35043 Marburg, Germany; 2 IQVIA, Epidemiology, Unterschweinstiege 2-14, 60549 Frankfurt am Main, Germany

**Keywords:** breast cancer, urinary incontinence, tamoxifen, aromatase inhibitor, depression, Germany

## Abstract

Purpose: Previous data showed an increased risk of developing urinary incontinence in breast cancer patients. However, there is a lack of studies including both pre and postmenopausal women. The aim of this study was to analyze the incidence of subsequent urinary incontinence in breast cancer patients and variables associated with an increased urinary incontinence. Methods: This study utilized IQVIA Disease Analyzer database to examine the five-year cumulative incidence of urinary incontinence among 68,066 women diagnosed with breast cancer in gynecological practices in Germany between January 2005 and December 2021 by using Kaplan–Meier curves, stratified by age group. Multivariable Cox regression models were conducted to assess the association between age, co-diagnoses, and endocrine therapy (tamoxifen, aromatase inhibitors) and incident urinary incontinence. Results: Within five years of the start of follow-up, 5.8% of women were diagnosed with urinary incontinence. Age (HR = 1.36; 95% CI = 1.21–1.54 for age 51–60; HR = 2.06; 95% CI = 1.84–2.31 for age group 61–70 and HR = 2.71; 95% CI = 2.42–3.04 for age group >70 as compared to age group ≤50), depression (HR = 1.41; 95% CI: 1.25–1.59) and menopausal and other perimenopausal disorders (HR = 1.21; 95% CI: 1.10–1.27) were associated with an increased urinary incontinence risk. The association was negative and statistically significant for aromatase inhibitor therapy (HR = 0.71; 95% CI: 0.66–0.78) as compared to women without endocrine therapy, whereas tamoxifen therapy was not associated with decreased or increased urinary incontinence risk. Conclusions: In conclusion, this study reports an 5.8% incidence of urinary incontinence within five years after breast cancer diagnosis. Age-related differences and co-diagnoses should be taken into account.

## 1. Introduction

Breast cancer is the most common malignant disease affecting women [1,2]. Due to efficient screening programs, modern cancer treatment, and structured follow-up programs, breast cancer survival rates have increased dramatically [3,4]. As more than two-thirds of malign breast tumors express estrogen and/or progesterone receptors [5,6], adjuvant hormone therapy has a special importance in breast cancer therapy and contributes remarkably to long-term survival of cancer patients [3,7]. Endocrine therapy comprises different agents, including tamoxifen, a selective estrogen receptor modulator (SERM) blocking estrogen effects on breast tissue, and aromatase inhibitors which suppress estrogen production from the adipose tissue [3,8]. Not only chemotherapy but also both tamoxifen and aromatase inhibitors can cause adverse symptoms due to estrogen deficiency, such as vasomotor symptoms, musculoskeletal disorders, reduced sleep quality and fatigue, psychosomatic disorders, and, moreover, urogenital complaints [3,8,9,10,11]. Not surprisingly, the effects of iatrogenic estrogen deficiency can be similar to the physiologically observed effects of aging and menopause on the lower urinary tract and may comprise nocturia, frequency, and urgency, including overactive bladder symptoms and urinary incontinence [12,13,14,15]. Nonetheless, the prolonged and higher degree of hypoestrogenism produced by breast cancer treatments can lead to more distinct pelvic floor symptoms [16], which can be associated with not only decreased health-related quality of life [17] but also depressive disorder [18,19]. As a breast cancer diagnosis itself has been associated with an increased risk of depressive symptoms [18], the coexistence of breast cancer and urinary incontinence may further exacerbate impairments in health-related quality of life, with potential negative consequences for social functioning, sexual health, and mental well-being [20].

As especially adjuvant endocrine therapy is usually applied for at least five years, reduced life quality caused by persisting side-effects may lead to non-compliance or even therapy discontinuation [3,8,21,22,23,24,25,26,27].

Therefore, we need to create awareness for the underlying mechanisms between endocrine breast cancer therapy, urological symptoms, and, moreover, mental health. Unfortunately, limited data exists comparing the effects of both tamoxifen and aromatase inhibitors on urinary incontinence. Moreover, there is a lack of studies including both pre and postmenopausal women. Consequently, the aim of this study is to analyze the five-year incidence of urinary incontinence in breast cancer patients in consideration of age, co-diagnoses, and adjuvant endocrine therapy with tamoxifen or aromatase inhibitors.

## 2. Materials and Methods

### 2.1. Database

The present study utilized data from the Disease Analyzer database (IQVIA). The database has already been described in the literature [28]. Briefly, the Disease Analyzer database contains anonymized sociodemographic, diagnosis, and prescription data collected in general and specialized practices in Germany. Practices to include in the database are selected using multiple criteria (i.e., physician age, practice specialty, size of the community, and federal state). Finally, the Disease Analyzer database has been found to be representative of German private practices [28] and has been widely used for studies on urinary incontinence [29,30].

### 2.2. Study Population

This study included women aged ≥ 18 years who were diagnosed for the first time with breast cancer (ICD-10 codes: C50) in one of 163 gynecological practices in Germany between January 2005 and December 2021 (index date). To be included in the analyses, women must not have had any urinary incontinence diagnoses (ICD-10 codes: R32, N39.3, N39.4) prior to the index date, on the index date, or within six months following the index date.

### 2.3. Study Outcome

The outcome of the study was the five-year incidence of urinary incontinence in women with breast cancer. Each patient was followed for up to five years, beginning on day 184 after the initial breast cancer diagnosis, until the earliest of either a first diagnosis of urinary incontinence or the last recorded visit in the practice. This follow-up strategy was necessary to allow for the identification of metastases, which are often diagnosed or documented within the first weeks or months following the initial breast cancer diagnosis; however, this approach may have excluded early urinary incontinence events occurring shortly after diagnosis

### 2.4. Study Covariates

Covariates were age (≤50, 51–60, 61–70, >70 years), availability of distant metastasis on index date or within six month after index date (ICD-10: C78, C79), and conditions diagnosed in at least 5% of study population including menopausal and other perimenopausal disorders (ICD-10: N95), inflammation of vagina and vulva (ICD-10: N76), leiomyoma of uterus (ICD-10: D25), noninflammatory disorders of ovary, fallopian tube and broad ligament (ICD-10: N83), depression (ICD-10: F32, F33), and anxiety disorder (ICD-10: F41). Finally, tamoxifen and aromatase inhibitor therapy were considered and defined as at least one documented prescription during the follow-up period. Information on dose, duration of therapy, treatment adherence, therapy switches, or concomitant use of gonadotropin-releasing hormone (GnRH) analogs was not available.

### 2.5. Statistical Analyses

Patient characteristics were reported by presenting continuous variables as mean values with standard deviations and categorical variables as absolute frequencies and proportions. The cumulative incidence of urinary incontinence during the five years from the start of follow-up was analyzed using Kaplan–Meier curves, stratified by age group. The association between age, distant metastasis, co-diagnoses, endocrine therapy (tamoxifen, aromatase inhibitors), and incident urinary incontinence was investigated with multivariable Cox regression models. The results of the Cox proportional hazards models are presented as hazard ratios (HRs) with corresponding 95% confidence intervals (CIs). No missing values were observed in the analyzed variables. Statistical significance was defined as a two-tailed *p*-value of less than 0.05. All statistical analyses were conducted using SAS software, version 9.4 (SAS Institute, Cary, NC, USA).

## 3. Results

### 3.1. Characteristics of the Population

There were 68,066 women with breast cancer included in the study. The characteristics of the population are displayed in Table 1. The mean (standard deviation) age was 60.6 (13.6) years. In 2.8% of women, distant metastasis was documented on index date or within six months after the index date. Overall, 17.5% of women received at least one prescription of tamoxifen and 22.5% received at least one prescription of an aromatase inhibitor during follow-up.

In terms of comorbidities, menopausal and other perimenopausal disorders (33.8%), inflammation of vagina and vulva (14.8%), leiomyoma of uterus (6.4%), noninflammatory disorders of ovary, fallopian tube, and broad ligament (5.3%), depression (6.9%), and anxiety disorders (9.3%) were the commonest with the proportion of at least 5% (Table 1).

### 3.2. Breast Cancer and Incident Urinary Incontinence

**Figure 1:**
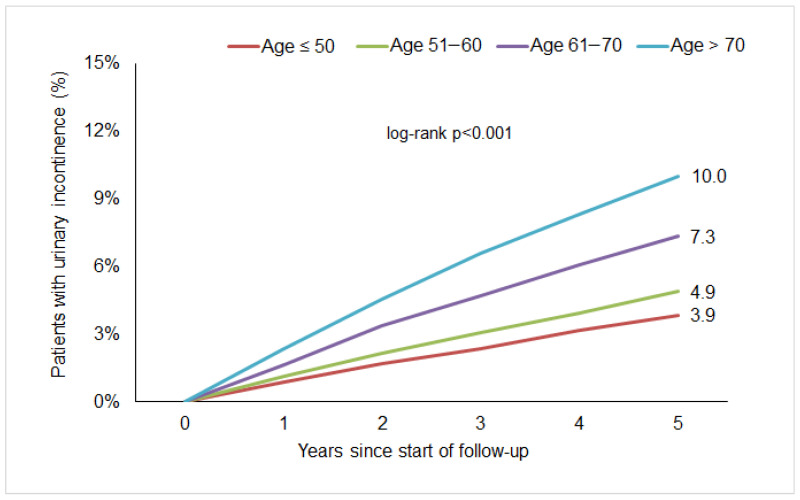
Five-year cumulative incidence of urinary incontinence in women with breast cancer followed in gynecological practices in Germany.

Within five years from the start of follow-up, 5.8% of women were diagnosed with urinary incontinence. Kaplan–Meier curves are displayed in Figure 1. There was 3.9% of the age group ≤50 years, 4.9% of the age group 51.60 years, 7.3% of the age group 61.70 years, and 10.0% of the age group >70 years.

### 3.3. Variables Associated with Urinary Incontinence in Breast Cancer Women

In the multivariable Cox regression analysis, higher age was positively and significantly associated with incident urinary incontinence (HR = 1.36; 95% CI = 1.21–1.54 for age 51–60; HR = 2.06; 95% CI = 1.84–2.31 for age group 61–70 and HR = 2.71; 95% CI = 2.42–3.04 for age group >70 as compared to age group ≤ 50, Table 2). Among co-diagnoses, the strongest association with urinary incontinence risk was observed for depression (HR = 1.41; 95% CI: 1.25–1.59). Menopausal and other perimenopausal disorders were also associated with an increased urinary incontinence risk (HR = 1.21; 95% CI: 1.10–1.27). Finally, the association was negative and statistically significant for aromatase inhibitor therapy (HR = 0.71; 95% CI: 0.66–0.78) as compared to women without endocrine therapy. Tamoxifen therapy was not associated with decreased or increased urinary incontinence risk, (Table 2).

## 4. Discussion

The aim of this study was to examine the 5-year incidence of urinary incontinence in 68,066 female breast cancer patients. Within five years after breast cancer diagnosis, 5.8% of women were diagnosed with urinary incontinence. Higher age was positively and significantly associated with incident urinary incontinence (Table 2). Furthermore, a co-diagnosis of depression showed the strongest association with urinary incontinence. In addition, menopausal and other perimenopausal disorders were associated with an increased urinary incontinence risk. The prescription of aromatase inhibitors showed a negative association with incident urinary incontinence in comparison with no endocrine therapy. Interestingly, tamoxifen therapy was not associated with increased or decreased urinary incontinence risk.

### 4.1. Incidence of Urinary Incontinence in Breast Cancer Patients

The present study shows a 5.8% incidence of urinary incontinence within five years following breast cancer diagnosis. In line with this finding, Milsom and Gyhagen estimate the mean annual incidence of urinary incontinence in the general population to range between 1% and 9%. Overall, urinary incontinence affects approximately 25% to 45% of women, although prevalence estimates vary considerably depending on the definitions used and the characteristics of the study population [31]. While urinary incontinence is generally defined as any involuntary leakage of urine, some studies differentiate between specific types—such as stress, urge, or mixed incontinence—or consider its frequency [31].

Focusing on breast cancer patients, this group tends to have higher prevalence of urinary incontinence in comparison to general population [17,19,32,33]. In a systematic review, Colombage et al. revealed a prevalence of urinary incontinence of 38% for breast cancer patients compared to 21% in healthy women [33]. In another study, Colombage et al. showed a difference of 17% regarding the occurrence of any type of urinary incontinence between women with and without breast cancer, even though they could not demonstrate statistically significant associations between breast cancer and the presence of incontinence after controlling for risk factors such as parity or obesity [32]. While some data indicate a high prevalence of urinary incontinence even prior to treatment initiation [24], its occurrence may further increase during the course of cancer therapy [3,24,34]. Nevertheless, the existence of shared risk factors has to be taken into account [24].

### 4.2. Association of Age and Incident Urinary Incontinence

In addition to obesity, age is a well-established contributor to urinary incontinence [35,36,37]. In general, the prevalence of urinary incontinence increases with age [12,31], a trend that is also evident among breast cancer patients [3,11,12,38]. In the present study, age was also positively and significantly associated with the risk of developing urinary incontinence (Table 2).

### 4.3. Endocrine Therapy and Risk of Urinary Incontinence

Beyond age-related factors, it is noteworthy to consider whether cancer therapy itself may contribute to an elevated risk of urinary incontinence. Approximately two of three malign breast tumors express estrogen and/or progesterone receptors [5,6], so that adjuvant hormone therapy is an important component in breast cancer therapy [3,7]. In this study, 40% of patients included received an endocrine therapy with tamoxifen (17.5%) or aromatase inhibitors (22.5%). Comparing the incidence of urinary incontinence in patients treated with and without endocrine therapy, a negative and statistically significant association was observed for the prescription of aromatase inhibitors; however, this finding may be influenced by residual confounding and indication bias. In contrast, tamoxifen therapy was not associated with decreased or increased urinary incontinence risk.

The definition of endocrine therapy exposure in the present study was necessarily simplified and based on documented prescriptions only. Detailed information on cumulative dose, duration of therapy, treatment adherence, therapy switches between tamoxifen and aromatase inhibitors, or combined use with GnRH analogs was not available. Therefore, potential differential effects related to treatment intensity or sequencing could not be assessed.

This finding is concordant with various studies demonstrating no association between urinary incontinence diagnosis and prescription of tamoxifen [37,39,40]. Leining et al. demonstrated no difference in bladder control between tamoxifen users and nonusers in both pre and postmenopausal women [40]. Morales et al. found no significant changes in urinary problems over a three-month period of tamoxifen intake in postmenopausal patients. Alfano et al. showed no significant association between urinary incontinence and tamoxifen use, but a trend for severe complaints in postmenopausal in comparison to premenopausal women [39].

In contrast to this, Veronesi et al. discovered more urinary disturbances in healthy women under preventive therapy with tamoxifen compared to a placebo group [41].

In agreement with this, Imamoglu et al. showed a strong relation between the presence of urinary incontinence and tamoxifen usage regardless of the duration of therapy, age, or menopausal status with a prevalence of almost 53% among tamoxifen takers [34]. Reasons for these contrasting findings may be the small sample size and the selection of the control group with a significantly lower rate of urinary incontinence as described in other studies [34].

Although both tamoxifen and aromatase inhibitors are summarized under endocrine therapy, their urogenital side-effects may differ. Therefore, studies that include both therapeutic branches are presented below.

Karaboyun et al. examined premenopausal breast cancer patients under at least six months therapy with tamoxifen and patients receiving aromatase inhibitors via a self-administered questionnaire. They revealed a statistically significant difference between the tamoxifen and aromatase inhibitor groups regarding newly developed urinary incontinence. Whereas more than 40% of patients under therapy with tamoxifen were diagnosed with urinary incontinence, this applied to only 15% of patients under therapy with aromatase inhibitors [3]. Ganz et al. reported an increase in bladder control problems in tamoxifen-treated patients, whereas there was no increase in women under therapy with aromatase inhibitors or without any endocrine therapy [11]. The effects of a combination with luteinizing hormone-releasing hormone (LHRH) receptor agonists, for example, when using aromatase inhibitors in premenopausal women, regarding urinary incontinence, are inconsistent [3,42].

The data presented suggest that different agents used to treat estrogen- and/or progesterone-receptor-positive breast tumors may be associated with different patterns of genitourinary symptoms. Endocrine therapy in general can cause adverse symptoms due to estrogen deficiency on every estrogen-sensitive tissue [3,8,9,10]. Tamoxifen, a selective estrogen receptor modulator (SERM), blocks estrogen effects on breast tissue [3,8]. In other tissues it can work as an agonist, whereas its role on the vagina and lower urinary tract is not completely clear and may even change with age [43]. Tamoxifen may increase the weight of the urethra and the thickness of the distal urethral epithelium [44]. It may increase the number of periurethral blood vessels and decrease their resistance leading to an improved blood supply in periurethral muscles [45,46]. SERMs may also support the thickness of the epithelial layer, collagen content, and muscle bundles of the bladder [34,47,48] leading to improved urinary continence. Nevertheless, tamoxifen may also have adverse effects by reducing the urethral resistance [34,45,49]. In addition, taking a possible shift in vaginal estrogen receptors with age into account, Fan and Zimmern suspect a partial agonistic effect of tamoxifen in postmenopausal women which may cause worsening of urinary incontinence [43,45]. However, this experimental research was performed using rat tissue or in vitro cell culture models and needs further clinical validation [34].

In contrast to SERMs, aromatase inhibitors suppress the estrogen production from the adipose tissue [3,8]. In premenopausal breast cancer patients ovarian suppression is added [42]. As some studies suggest severe bladder complications under prescription of tamoxifen in contrast to aromatase inhibitors [3,11], Landi et al. suppose that—as oral menopausal hormone therapy with estrogen can be associated with an increased risk for urinary incontinence—higher estrogen levels under therapy with tamoxifen compared to aromatase inhibitors can be one explanation for a higher risk for urinary incontinence [50]. Dose-dependent effects of estrogen on, for example, the urethral resistance, are discussed as one reason for the inconsistent findings regarding incontinence under endocrine therapy [45].

However, a wide range of studies could not find any significant differences in urinary incontinence symptoms between breast cancer patients treated with different types of endocrine therapy [9,12,42,50].

Two studies comparing the side-effects of endocrine therapy with tamoxifen with or without ovarian suppression and aromatase inhibitors with ovarian suppression in premenopausal patients did not find different rates of incontinence [42]. Stahlschmidt et al. compared women treated with tamoxifen and aromatase inhibitors and found no differences in the symptom burden regarding stress urinary incontinence or overactive bladder. Moreover, there was no association with the presence of the latter and duration of treatment. Interestingly, there was an association between incomplete adherence to endocrine therapy and severity of urinary symptoms [12].

In a prospective study with postmenopausal patients, Baumgart et al. found no significant difference in urinary incontinence symptoms between breast cancer patients treated with tamoxifen or aromatase inhibitors and a healthy control group [9]. Consistent with this finding, Landi et al. found no significant differences in newly diagnosed urinary incontinence in patients under endocrine therapy with tamoxifen or aromatase inhibitors and breast cancer patients without endocrine therapy adjusted for age, time since diagnosis, race, parity, smoking status, hysterectomy history, and BMI [50].

Not statistically significant data suggested an increased risk among tamoxifen users and a decreased risk for users of aromatase inhibitors [50]. Similar to this finding, the present study observed not only no association between tamoxifen therapy and incident urinary incontinence but also a negative and statistically significant association for the prescription of aromatase inhibitors and urinary incontinence risk. However, due to the observational and retrospective nature of the present study, these findings reflect associations only and do not allow conclusions regarding causal effects of endocrine therapy on urinary incontinence.

In particular, aromatase inhibitors are predominantly prescribed to postmenopausal women and to patients with specific tumor characteristics and recurrence risk profiles. Although age was adjusted for in the multivariable models, residual confounding related to age distribution, treatment indication, disease characteristics, or unmeasured lifestyle factors cannot be excluded. Given that higher age represents the strongest risk factor for urinary incontinence in the present study, the observed negative association should not be interpreted as a protective effect of aromatase inhibitors.

### 4.4. Relevant Co-Diagnoses in Breast Cancer Patients with Urinary Incontinence

In the present study, incident urinary incontinence in women with breast cancer was significantly associated with menopausal and other perimenopausal disorders.

The majority of patients included in this study were peri or postmenopausal. The recording of related symptoms such as hot flashes, musculoskeletal pain, or sleep disorders in this sample is easy to assume. An aggravation of (peri)menopausal symptoms can be associated with endocrine therapy, but also with chemotherapy toxicity [11], as both endocrine therapy and ovarian suppression caused by chemotherapy contribute to estrogen deficiency. As associations between altered estrogen levels and bladder control problems have been documented previously [3,8,9,10], and the observed association between incident urinary incontinence and menopausal and other perimenopausal symptoms in women with breast cancer appears plausible but remains non-causal.

In addition to physical wellbeing, mental health of cancer patients should not be neglected. An association between breast cancer diagnosis and depression [18] as well as an association between urinary incontinence and depressive disorder in cancer patients is discussed [18,19]. Beyond that, the present study showed a significant association between incident urinary incontinence and pre-existing depression in women with breast cancer. An explanation for this finding may be that patients with pre-existing depression are likely to be under antidepressant medication. Urinary incontinence can be a possible side-effect of commonly prescribed antidepressants such as selective serotonin reuptake inhibitors [51]. As breast cancer diagnosis increases psychological stress, increased dosage and, consequently, increased side-effects of antidepressant medication may explain the association of incident urinary incontinence and pre-existing depression in breast cancer patients.

Even though an interaction between breast cancer, urinary incontinence, and reduced mental health having an adverse influence on the quality of life when occurring simultaneously can be assumed, there is a lack of documentation of genitourinary syndrome, for example, regarding an increase in frequency or incontinence, in breast cancer survivors and, in addition to this, a lack of treatment plans [10,52,53]. Information about urinary symptoms associated with cancer and its treatment as well as access to screening and therapy can meet up to the present moment “unmet survivorship needs”. It can support cancer therapy adherence, contribute to life quality of breast cancer patients and even influence their life expectancy by preventing treatment discontinuation due to side-effects [52].

### 4.5. Strengths and Limitations

This retrospective study has several strengths, including the large sample size, a follow-up period of up to five years, the diagnosis of urinary incontinence by gynecological specialists, the inclusion of both pre and postmenopausal women, and the adjustment for important confounders such as age and a broad range of comorbidities. Nevertheless, the present study must also be interpreted in light of several limitations.

First, the identification of patients and outcomes based on ICD codes implies that the effects of incorrect coding or changes in coding practices over time cannot be excluded. In addition, further differentiation of urinary incontinence (e.g., stress, urge, or mixed incontinence) and information on symptom severity were not available. Furthermore, due to the retrospective cohort design and reliance on ICD-coded diagnoses, causal relationships cannot be inferred, and the observed associations may be influenced by residual confounding.

In this study, only 40% of the included patients received therapy with tamoxifen or aromatase inhibitors, which is lower than expected given that more than two-thirds of breast tumors express estrogen and/or progesterone receptors. This proportion may be explained by incomplete prescription documentation in gynecological practices, initiation or continuation of endocrine therapy in oncological settings not captured in the database, contraindications, early treatment discontinuation, or missing information on hormone receptor status.

Moreover, no information was available on tumor stage, tumor subtype, or the exact treatment algorithm. Therefore, differentiation between the effects of chemotherapy, ovarian suppression, and endocrine therapy was not possible. In addition, indication bias may have affected the observed associations between endocrine therapy type and urinary incontinence, as treatment allocation was not random and depended on menopausal status, age, and clinical characteristics that could not be fully controlled for.

Important confounders for urinary incontinence, such as body mass index, parity and mode of delivery, prior pelvic surgery, pelvic radiotherapy, type of breast surgery, and chemotherapy use, could not be considered. These variables are either not available or only rarely documented in outpatient gynecological electronic medical records captured in the Disease Analyzer database. In particular, body mass index is inconsistently recorded in routine gynecological documentation, and parity-related information is not systematically available. The lack of adjustment for these factors may have resulted in residual confounding and should be considered when interpreting the observed associations.

Finally, follow-up began 184 days after the initial breast cancer diagnosis. Although this approach was chosen to reduce misclassification related to early metastatic disease, it may have led to the exclusion of patients who developed urinary incontinence shortly after diagnosis or during early cancer treatment. Consequently, a degree of selection bias and survivorship bias cannot be excluded, and the reported incidence of urinary incontinence may be underestimated.

## 5. Conclusions

In conclusion, this study reports a 5.8% incidence of urinary incontinence within five years after breast cancer diagnosis. Age-related differences as well as therapy-specific associations should be taken into account; however, the observed negative association for aromatase inhibitors should be interpreted with caution due to the likelihood of residual confounding and indication bias. Associations of urinary incontinence in breast cancer patients and (peri) menopausal symptoms as well as depression were observed. Information about and adequate therapy of urinary incontinence is one unmet need of breast cancer patients.

## Figures and Tables

**Table 1:** Characteristics of women with breast cancer.

Variable	N (%)
N	68.066
Age (mean, SD)	60.6 (13.6)
Age ≤ 50	16,737 (24.6)
Age 51–60	16,432 (24.1)
Age 61–70	17,290 (25.4)
Age > 70	17,607 (25.9)
Distant metastasis	1919 (2.8)
Menopausal and other perimenopausal disorders	22,987 (33.8)
Inflammation of vagina and vulva	10,086 (14.8)
Leiomyoma of uterus	4376 (6.4)
Noninflammatory disorders of ovary, fallopian tube, and broad ligament	3573 (5.3)
Depression	4717 (6.9)
Anxiety disorders	6303 (9.3)
Prescriptions of tamoxifen	11,901 (17.5)
Prescription of aromatase inhibitors	15,325 (22.5)

Data are absolute numbers (percentages) unless otherwise specified.

**Table 2:** Association between defined variables and incident urinary incontinence in women with breast cancer.

Variable	Hazard Ratio (95% Confidence Interval)	*p*-Value
Age ≤ 50	Reference	
Age 51–60	1.36 (1.21–1.54)	<0.001
Age 61–70	2.06 (1.84–2.31)	<0.001
Age > 70	2.71 (2.42–3.04)	<0.001
Distant metastasis	1.18 (0.90–1.54)	0.232
Menopausal and other perimenopausal disorders	1.18 (1.10–1.27)	<0.001
Inflammation of vagina and vulva	0.99 (0.90–1.09)	0.825
Leiomyoma of uterus	0.91 (0.79–1.05)	0.193
Noninflammatory disorders of ovary, fallopian tube, and broad ligament	0.99 (0.84–1.17)	0.911
Depression	1.41 (1.25–1.59)	<0.001
Anxiety disorders	0.88 (0.72–1.04)	0.079
Prescriptions of tamoxifen	0.99 (0.91–1.08)	0.851
Prescription of aromatase inhibitors	0.71 (0.66–0.78)	<0.001
No endocrine therapy	Reference	

Hazard ratios and 95% confidence intervals were obtained using Cox regression models with urinary incontinence as the dependent variable, and age group, distant metastasis, endocrine therapy, and co-diagnoses as their dependent variables.

## Data Availability

Due to the proprietary nature of the Disease Analyzer database and IQVIA’s terms of data use agreement, research data cannot be shared. The data and the code used for this study are available from the corresponding author upon reasonable request.
